# Supplementary material for: Robust triboelectric energy harvesters engineered from electrochemically deposited films of HKUST-1 polycrystals
Source: Commun Chem. 2026 Feb 25;9:144. doi: 10.1038/s42004-026-01949-0 (PMC13046843; doi:10.1038/s42004-026-01949-0)
Supplement: Supplementary file 2 — SUPPLEMENTAL MATERIAL_pdf [file 42004_2026_1949_MOESM2_ESM.pdf]

Supplemental Information

Robust triboelectric energy harvesters engineered from electrochemically deposited films of HKUST-1 polycrystals

Chuzhan Jin and Jin-Chong Tan[*]

[a]Multifunctional Materials & Composites (MMC) Laboratory, Department of Engineering Science, University of Oxford, Parks Road, Oxford OX1 3PJ, U.K.

[*]Corresponding Author:
jin-chong.tan@eng.ox.ac.uk

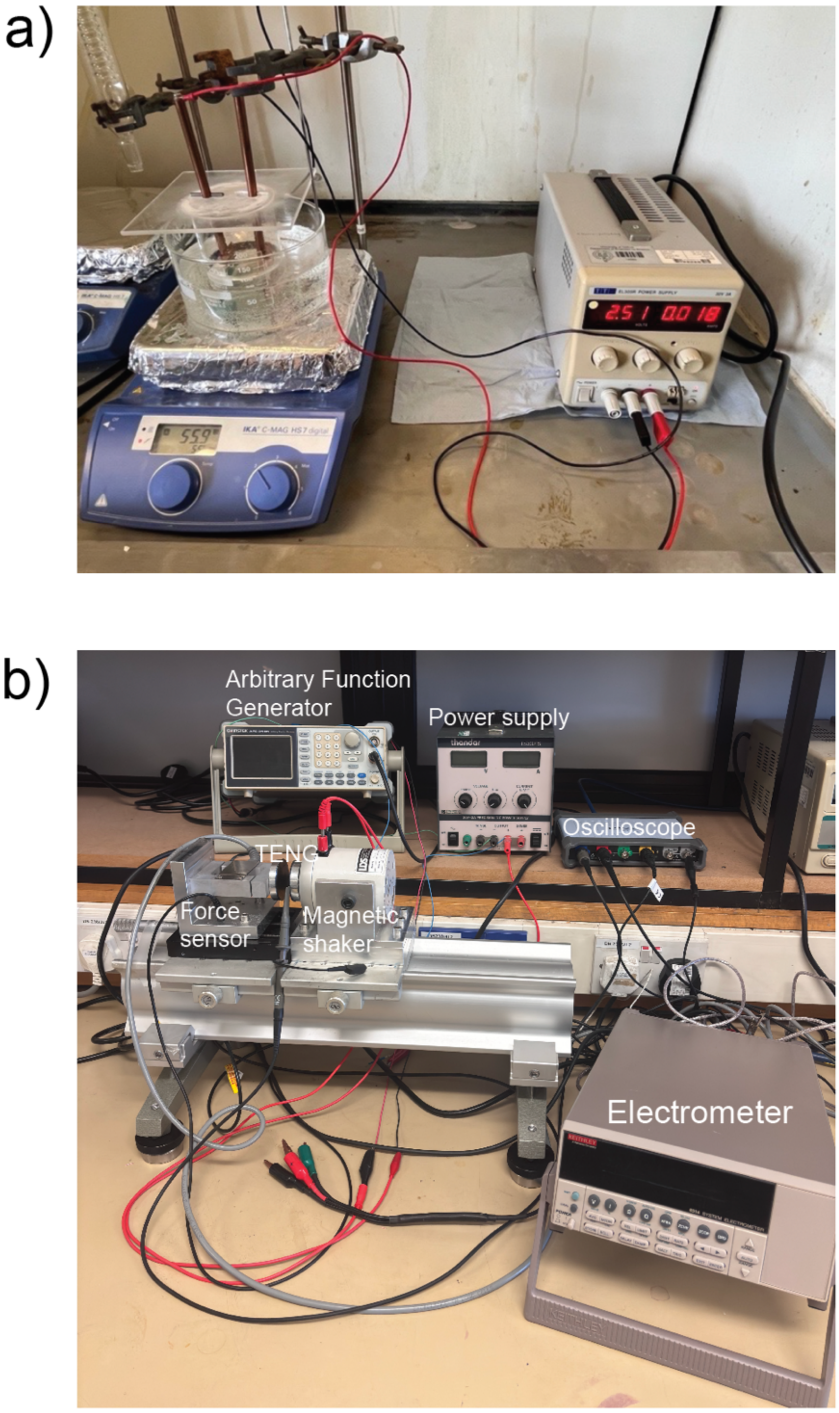

Supplementary Figure 1. a) Photograph of electrochemical deposition setup for growing the HKUST-1 MOF polycrystalline films. b) Photo of TENG set-up under contact-separation mode.

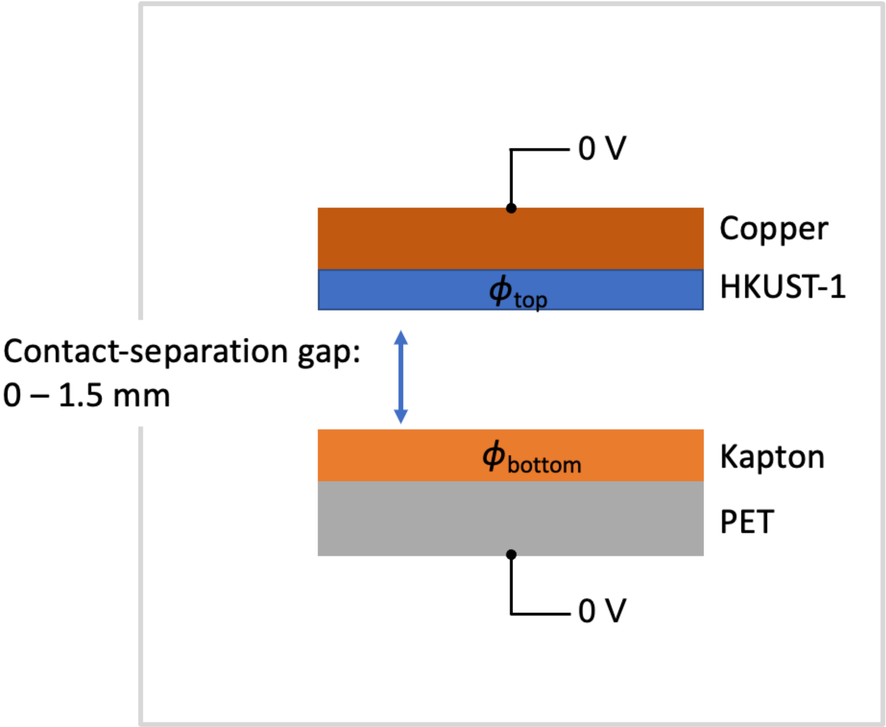

Supplementary Figure 2. Diagram of the geometry (not drawn to scale) of the finite-element model formulated in the COMSOL software. The TENG model (30 mm width × 30 mm depth) is enclosed within the 100 mm × 100 mm rectangular domain of the electrostatic simulation. Both the top and bottom electrodes (Copper/PET) have a constant ground potential of 0 V.

Supplementary Table 1. Parameters used in COMSOL simulation.

Layer	Thickness (mm)	Width (mm)	Relative permittivity	Surface charge density (C/m^2)
Copper	0.9	30	1	-
HKUST-1	0.06	30	10, 20, 30, 40, 55.73	1.09×10^{-5} (calculated)
Kapton	0.07	30	3.47	-1.09×10^{-5}
PET	1	30	2.9	-

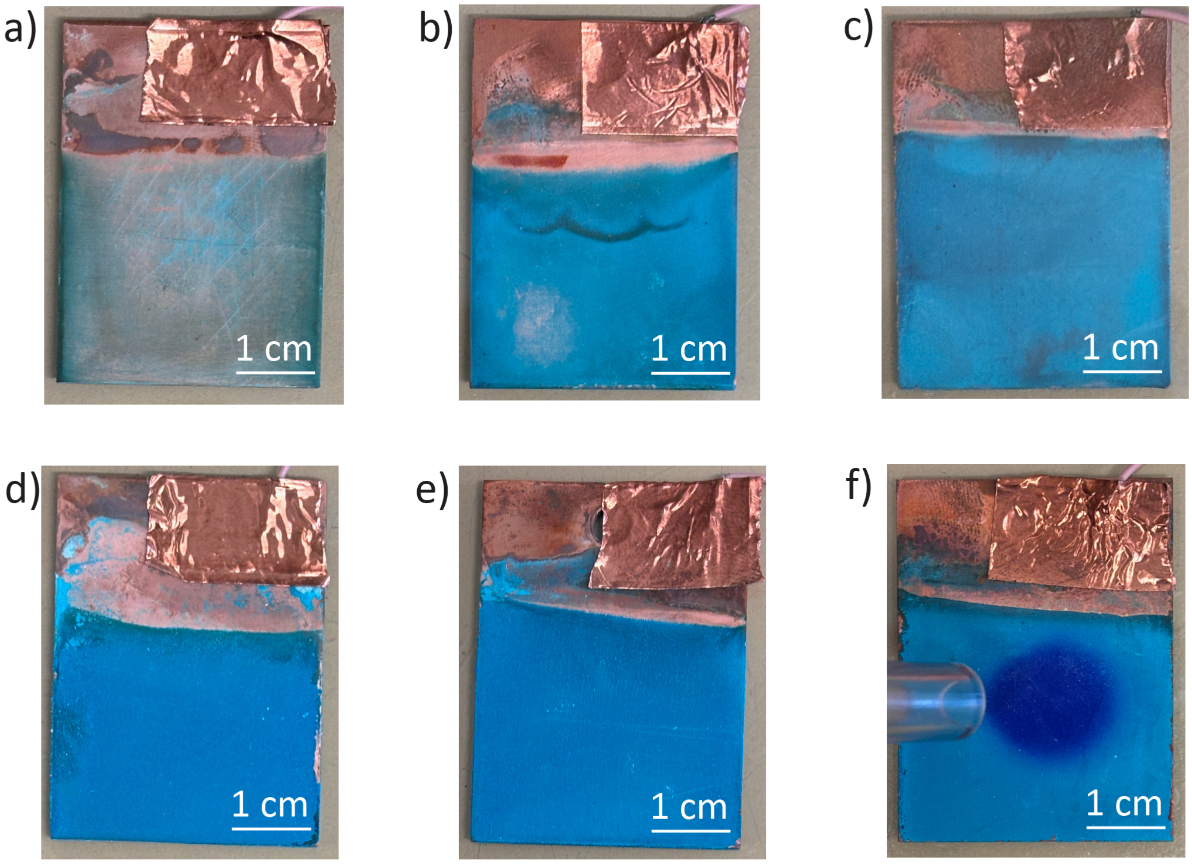

Supplementary Figure 3. HKUST-1 with different growth times at a) 0.25 h; b) 0.5 h; c) 1 h; d) 2 h; e) 3 h; and f) Dry N_2 gas on 2 h-HKUST-1 sample for reduced humidity.

Supplementary Table 2. Average thickness values taken from four different locations on each piece of electrodeposited sample with a specified growth time. The net film thickness was estimated by subtracting thickness of the substrate (growth time = 0) from the combined thickness measured at specified growth time.

Growth time / h	Combined thickness of substrate + polycrystalline film / mm	Net film thickness / μm
0 (substrate only)	0.911 ± 0.001	0
0.5	0.925 ± 0.004	14
1	0.933 ± 0.001	22
2	0.951 ± 0.005	40
3	0.976 ± 0.009	65

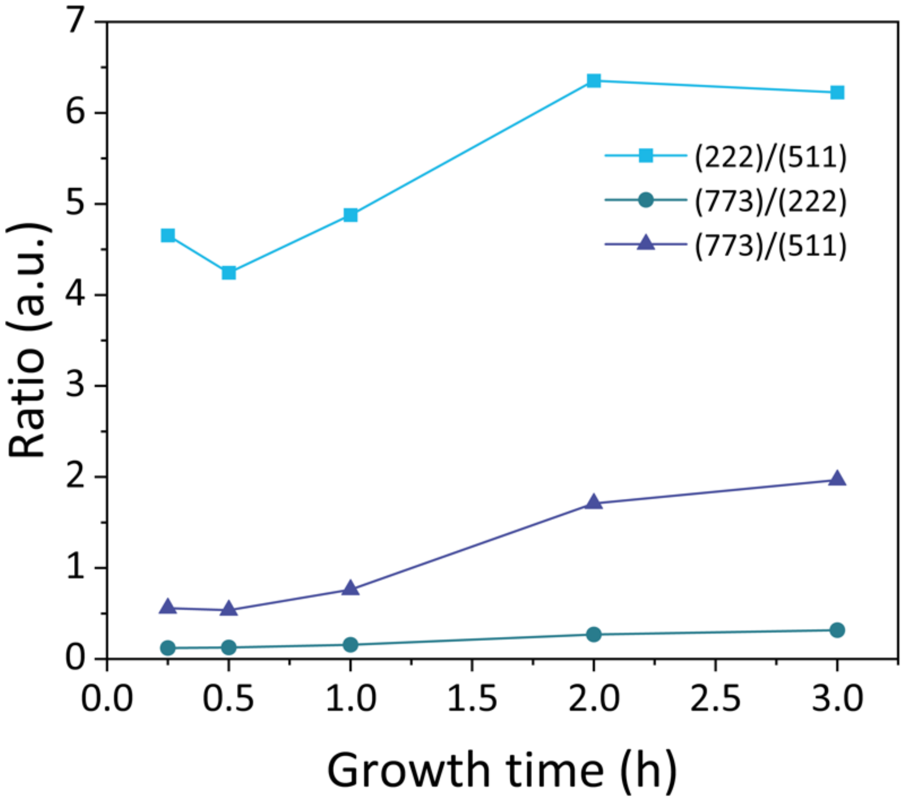

Supplementary Figure 4. The peak ratio from three intense peaks calculated in 5 different growth times based on the XRD spectra, showing the improvement in crystallinity of the HKUST-1 polycrystalline films.

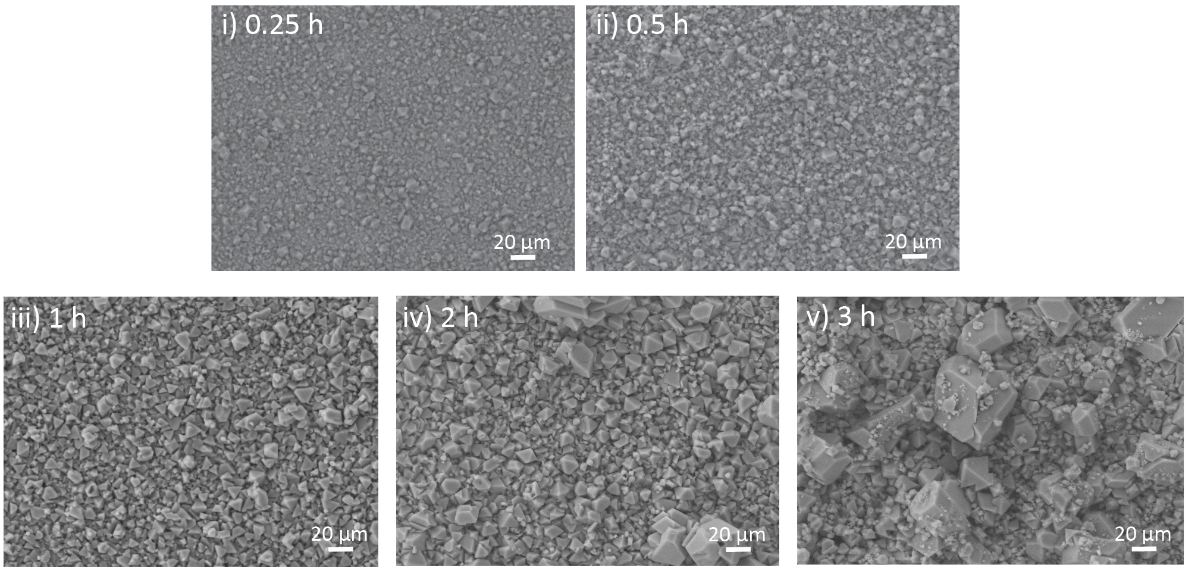

Supplementary Figure 5. Different growth time i) 0.25 h; ii) 0.5 h; iii) 1 h; iv) 2 h; v) 3 h for HKUST-1.

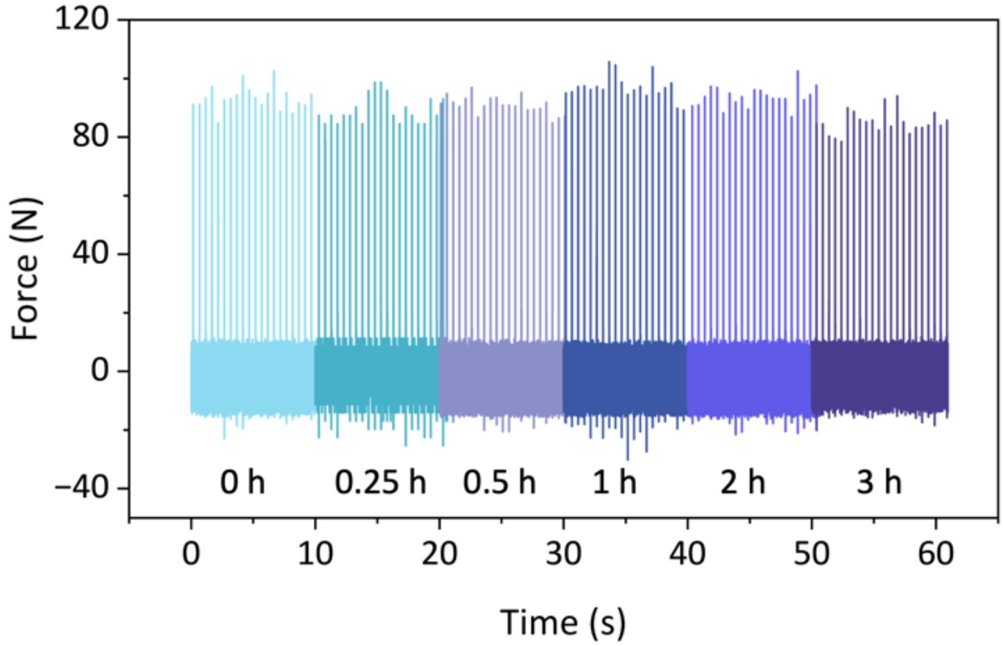

Supplementary Figure 6. Force measurement during HKUST-1 TENG output performance.

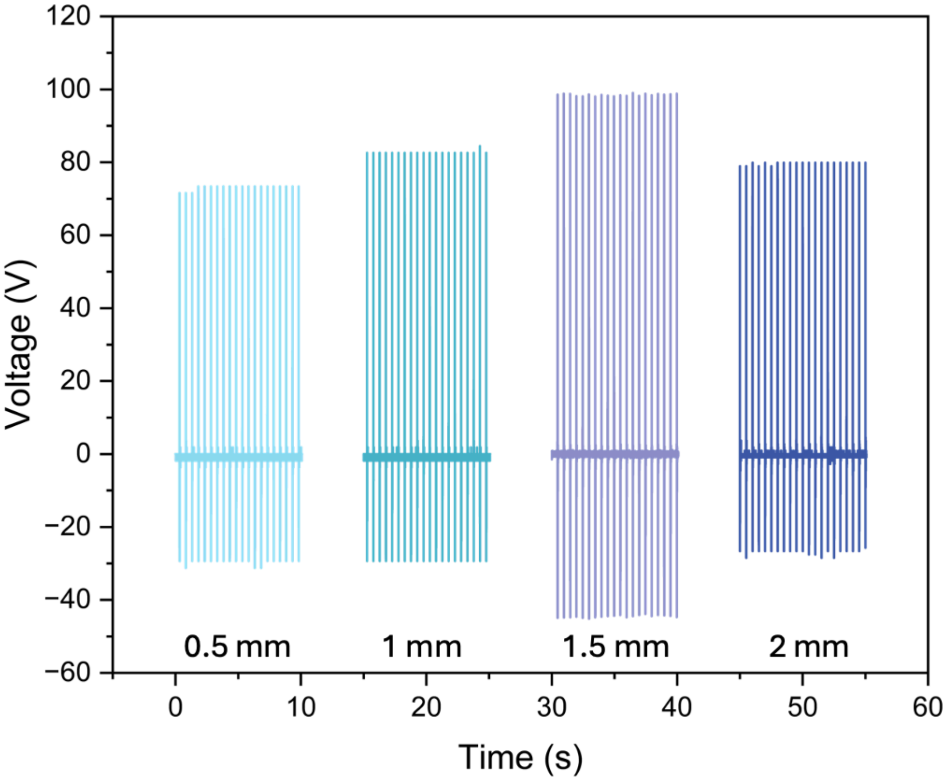

Supplementary Figure 7. Voltage output with different distance between HKUST-1 and Kapton.

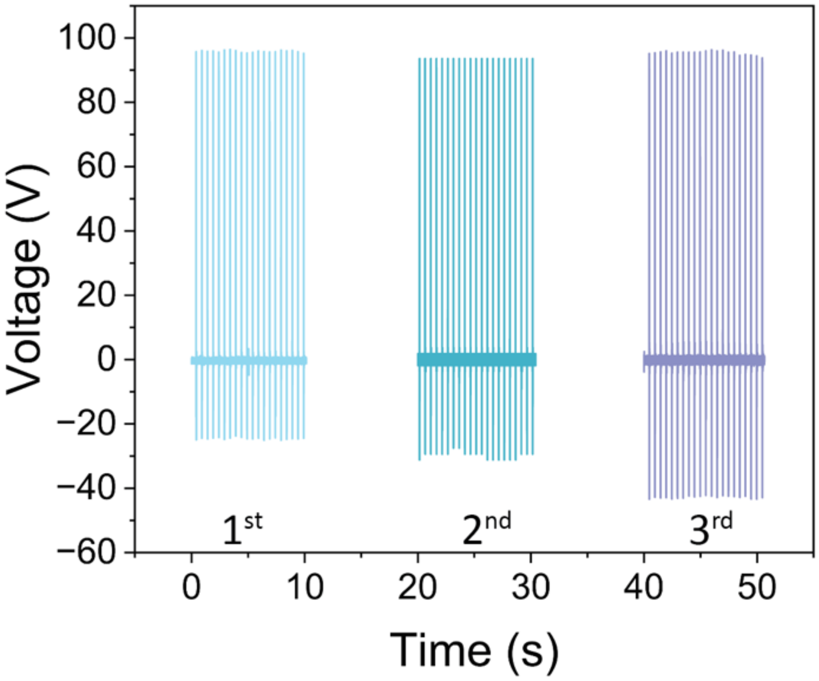

Supplementary Figure 8. Reproducibility shown from three batches of samples, with an average voltage of 95.4 ± 1.6 V.

**Preparation method**	**Tribo positive layer**	**Tribo negative layer**	**Area (cm^2)**	**Power density ($mW\ m^{-2}$)**	**Voltage (V)**	**Current (μA)**	**Current density ($\mu A\ cm^{-2}$)**	**Reference**
Immersion at room temperature (RT) with different cycles	ZIF-8 on ITO-PET	Kapton with copper and PET	2.5 × 2.5	392	164	7	1.12	[1]
Immersion at RT for 24 h	ZIF-8 on wood	PDMS on wood	3.5 × 2		24.3	0.32	0.05	[2]
Hydrothermal method in 2 steps (~37h preparation time)	ZIF-67 on Al	FEP on Al	4.5 × 4.5	2350	280	70	3.46	[3]
Hydrothermal method at 100°C for 48 h	MOF-303 on Al	FEP on Al	4 × 4	7400	435	85	5.31	[4]
Solvothermal in-situ method at 120°C for 24 h	Cu foil	UiO-66-H on FTO glass substrate	1 × 1		7.46	0.07	0.07	[5]
	Cu foil	UiO-66-NH_2 on FTO glass substrate	1 × 1		5	0.05	0.05	[5]
	Cu foil	UiO-66-NO_2 on FTO glass substrate	1 × 1		23.79	0.29	0.29	[5]
	Cu foil	UiO-66-Br on FTO glass substrate	1 × 1		12	0.14	0.14	[5]

Supplementary Table 3. Comparison of direct growth of MOF-TENG.

Preparation method	Tribo positive layer	Tribo negative layer	Area (cm^2)	Power density ($mW\ m^{-2}$)	Voltage (V)	Current (μA)	Reference
Adhere on Al tape	ZIF-7	Kapton	2.5 × 2.5	392	60	1.1	6
	ZIF-9	Kapton	2.5 × 2.5		~28	~0.3	6
	ZIF-11	Kapton	2.5 × 2.5	2350	~26	~0.25	6
	ZIF-12	Kapton	2.5 × 2.5		~42	~0.6	6
Adhere on Al tape	ZIF-62	Teflon	2.5 × 2.5	9.68	62	1.4	7
Adhere on Cu tape	ZIF-67	Teflon	S-TENG	150	118	1.7	8
Adhere on Cu tape	ZIF-8	Kapton	3-unit TENG		150	4.95	9
Adhere on Al tape	MIL-88A	FEP	2.5 × 2.5	10.4	80	2.2	10
Adhere on Cu tape	Cyclodextrin (CD)-MOF	Teflon	Z-shape TENG	80	152	1.2	11
Adhere on Al tape	MOF-5	PTFE	2.5 × 2.5		484	40	12
Adhere on Cu tape	ZUT-8	PVDF	5 × 5	2217.18	562.78	97.03	13

Supplementary Table 4. Comparison of adhesion method of MOF-TENG.

Preparation method	Tribo positive layer	Tribo negative layer	Area (cm^2)	Power density ($mW\ m^{-2}$)	Voltage (V)	Current (μA)	Reference
Adhere on PET	ZTF-8	PTFE	2.5 × 2.5	720	293	13.5	[14]
Adhere on PET	ZIF8@ZIF67	Teflon	2.5 × 2.5	736	359	11.7	[15]
Adhere on Cu tape	Cd-MOF	PVDF	5 × 5	2451.04	451.8	55.32	[16]
Adhere on Cu tape	Zn/Co-MOF	Kapton	5 × 5	14480	611.78	58.19	[17]
Adhere on Cu tape	ZUT-75 (Co)	PVDF		3280.5	677.09	148	[7,18]
	ZUT-75 (Zn)	PVDF		2178	389.03	54.75	[7,18]
	ZUT-75 (Cu)	PVDF		1320.98	339.28	48.82	[7,18]
	ZUT-75 (Mn)	PVDF		1211.04	399.88	61.28	[7,18]
Blade-coated to Al tape	MIL-101 (Cr)-PY	PTFE	2.5 × 2.5	2503	830	11	[19]

Supplementary Table 5. Comparison of adhesion method of MOF-TENG.

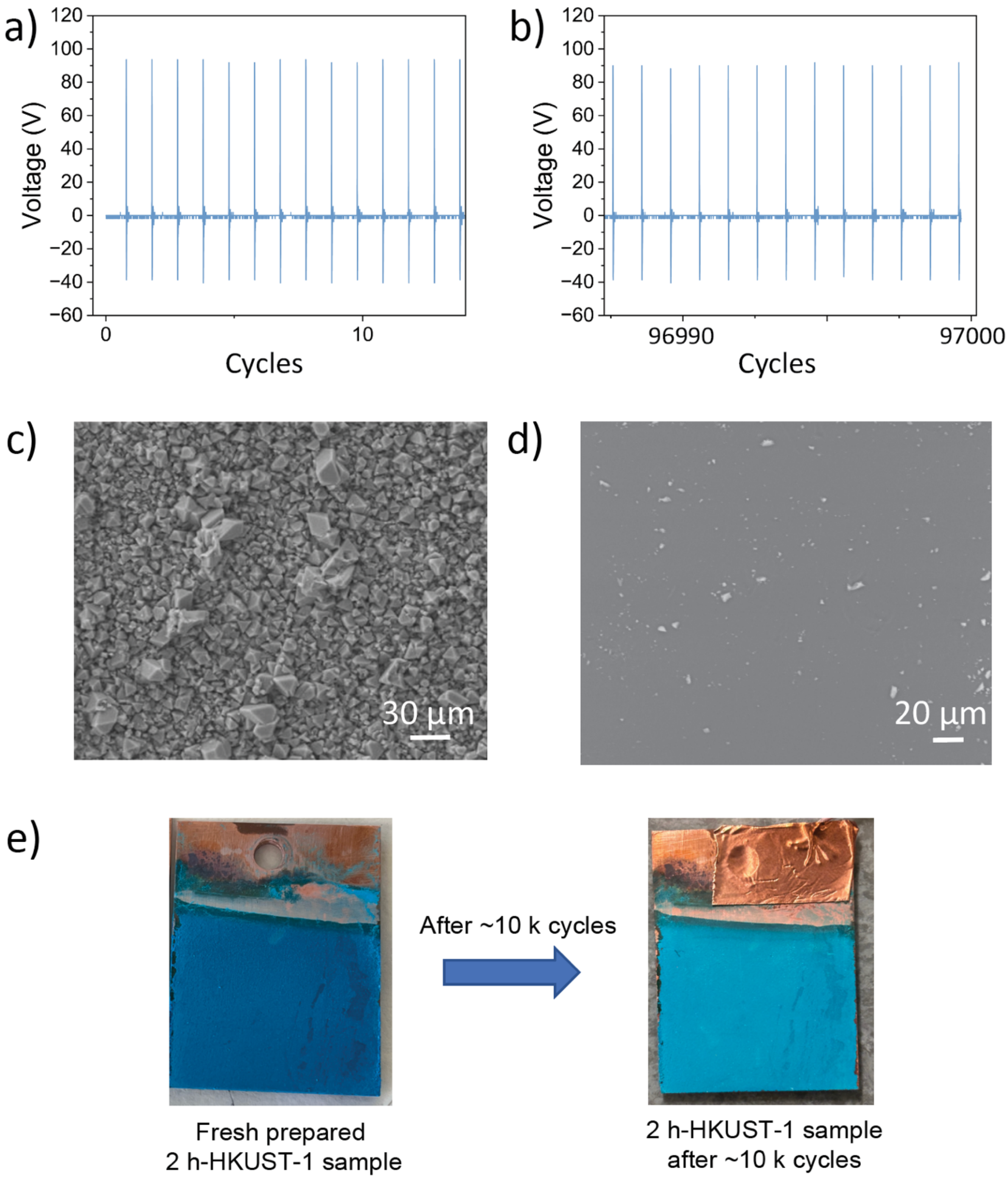

Supplementary Figure 9. a) Magnified view of representative cycles at the beginning and b) end of stability test. c) HKUST-1 image with similar area monitored before stability test. d) HKUST-1 crystal observed on Kapton tape after stability test. e) Photos taken before and after ~10,000 cycles tested in contact-separation motion. The color change was due to the uptake of moisture.

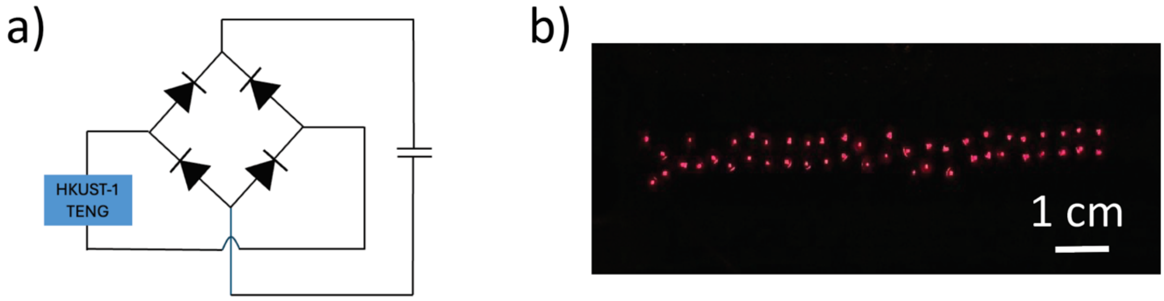

Supplementary Figure 10. a) Circuit diagram used for capacitor charging. b) Illumination of 48 LEDs by employing the electricity generated from the 2 h-HKUST-1 TENG device.

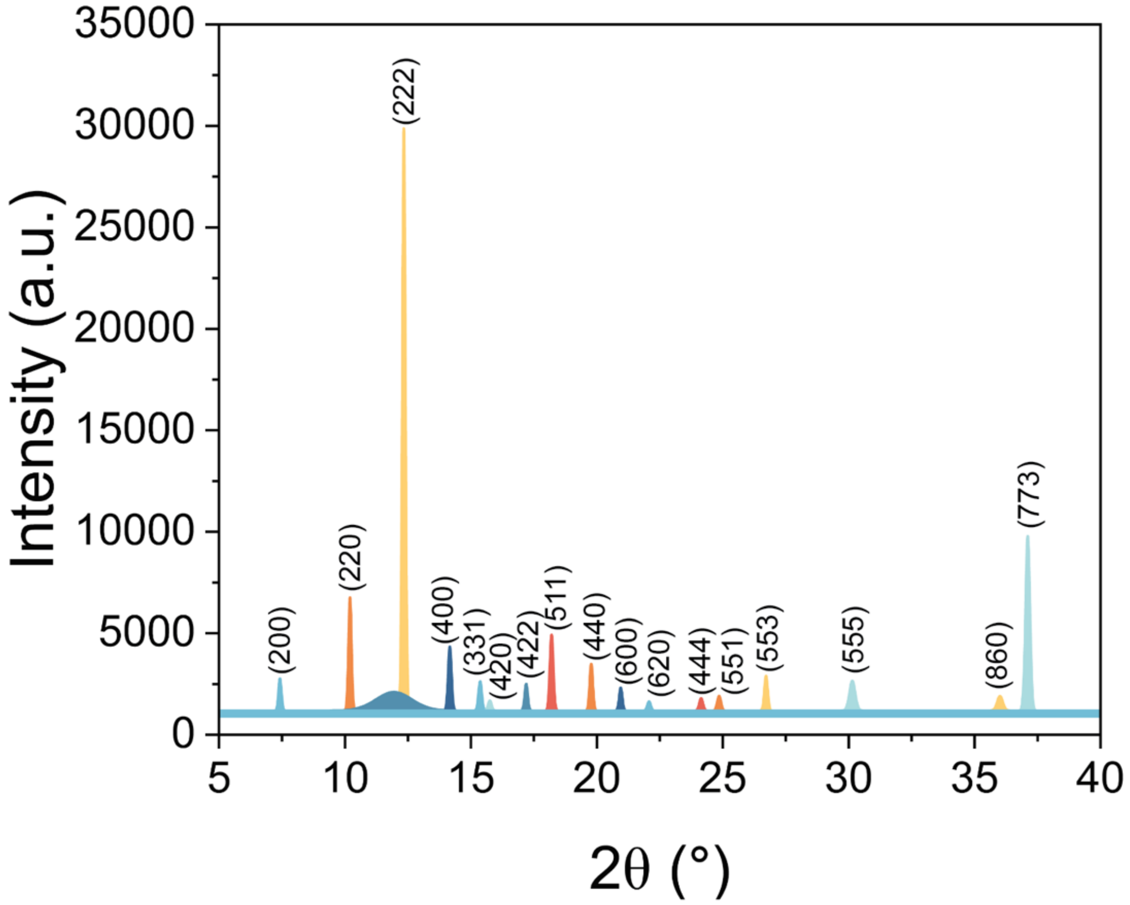

Supplementary Figure 11. Integrated peak areas highlighted in different colours for distinctive facets of HKUST-1.

Supplementary Table 6. Integrated areas, Bragg peak heights after baseline correction, and relative intensity of the 10 strongest reflexions in Figure S11, as calculated by (peak area / total peak area) × 100%.

Miller index (*hkl*)	2θ / °	Area Integration / $(au)^2$	Max. Height / au	Relative intensity (%)
**(222)**	12.34	4406.8	28692.3	35.6
**(773)**	37.11	1870.1	8596.2	15.1
**Peak from copper substrate**	11.94	1624.2	912.9	13.1
**(220)**	10.20	832.2	5570.9	6.7
**(511)**	18.20	634.6	3735.0	5.1
**(400)**	14.16	519.5	3147.3	4.2
**(555)**	30.14	419.4	1467.3	3.4
**(440)**	19.78	362.9	2295.7	2.9
**(553)**	26.72	282.3	1714.1	2.3
**(331)**	15.37	236.7	1434.7	1.9
**(200)**	7.42	228.2	1575.7	1.8

Peak integration was carried out using the Fit Peaks module in OriginPro 2025. The PXRD pattern of the 2 h HKUST-1 sample was baseline-treated with the constant baseline mode (minimum y = 852). The 10 strongest diffraction peaks were selected, including a minor hump from the copper substrate (Supplementary Figure 11). Integrated areas and relative intensities were listed in Supplementary Table 6.

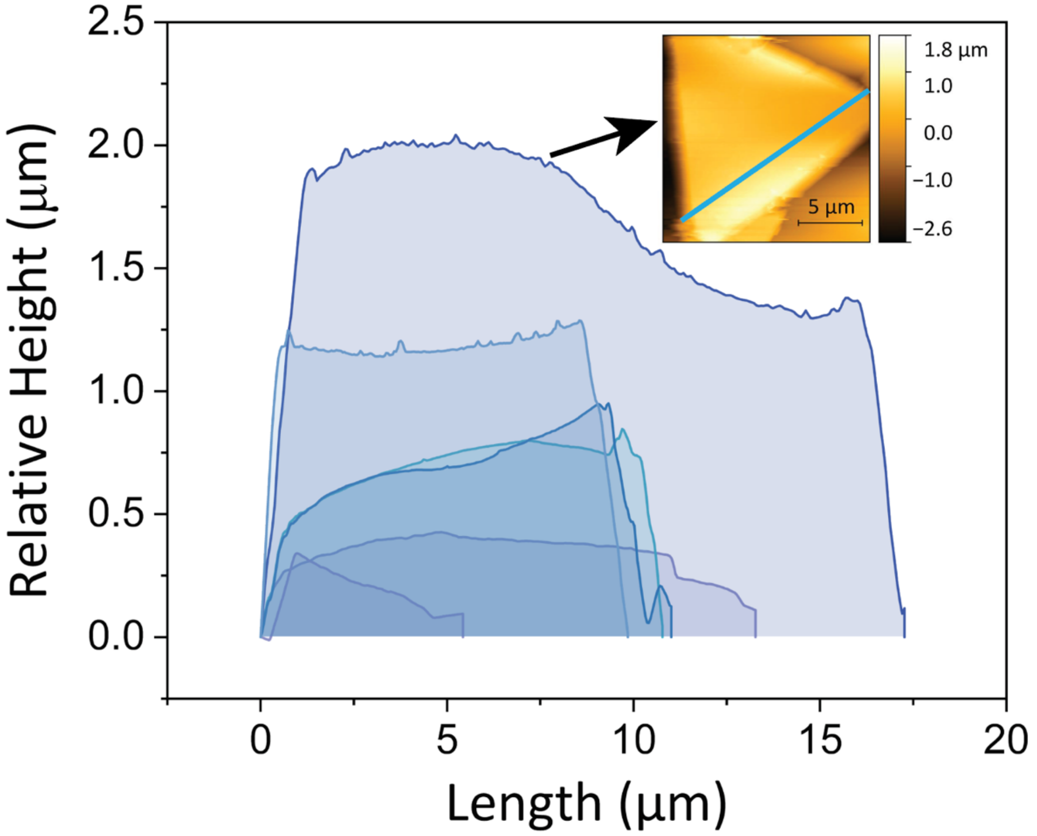

Supplementary Figure 12. Selected relative height difference on 2 h-HKUST-1 sample with inset of an example showing how these heights extracted from AFM height image.

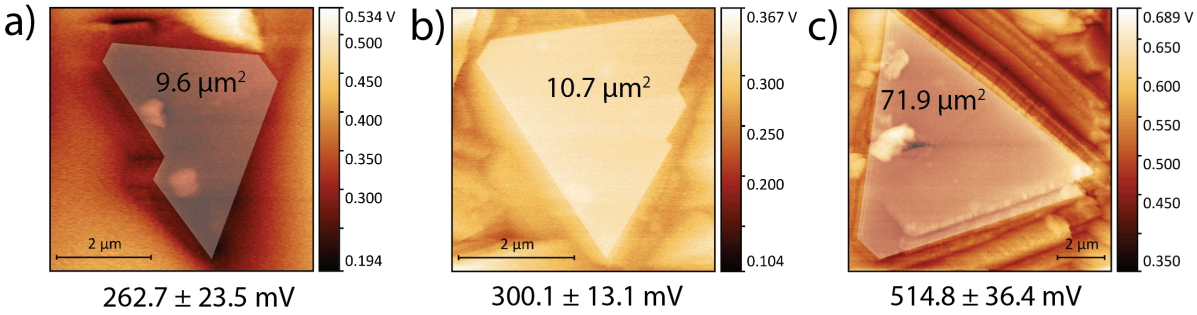

Supplementary Figure 13. KPFM surface potential images of the (222) facets. HKUST-1 crystals chosen after electrochemical deposition for a) 2 h, b) 0.5 h, and c) 1 h. Individual facets were masked (white) to calculate the average surface potentials on each facet.

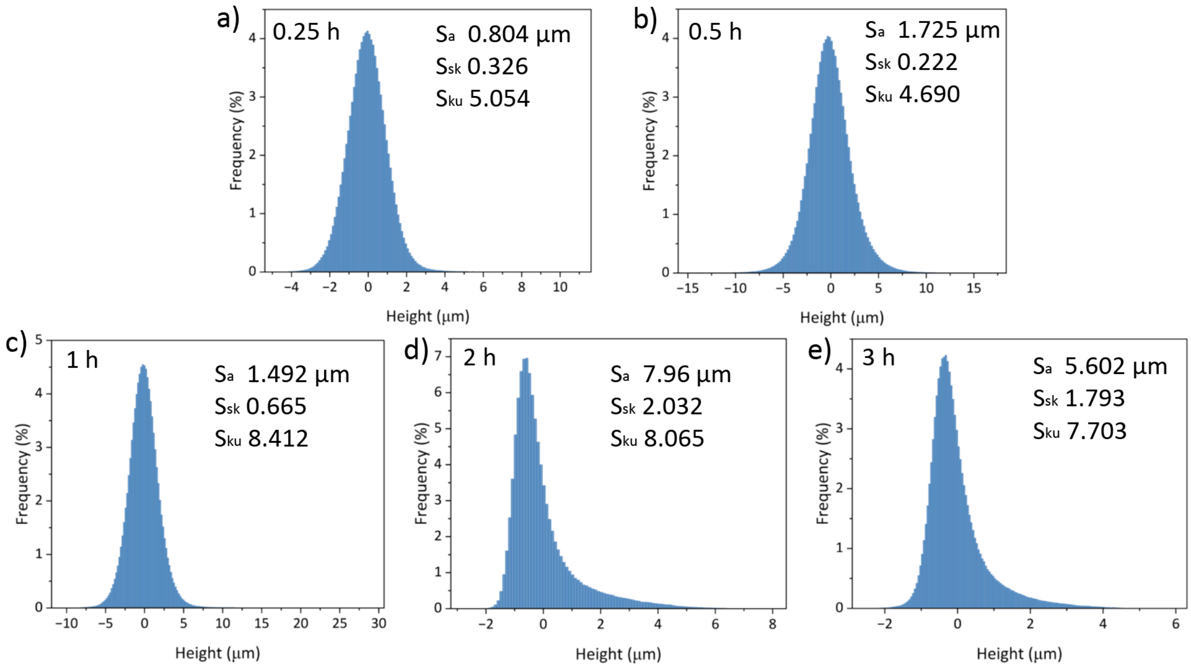

Supplementary Figure 14. Height distribution for the sample grown for 0.25 h, 0.5 h, 1 h and 3 h with the roughness measurements S_a (Mean height deviation), S_{sk} (Skewness), S_{ku} (Kurtosis) obtained from the Alicona optical profilometer.

Supplementary Table 7. Area factor, S_{dr}, calculation for each growth time of the HKUST-1 polycrystalline film by electrodeposition.

Growth time / h	Area with texture / µm^2	S_{dr} / %
0.25	25.2	0.6
0.5	25.7	2.8
1	25.5	2.0
2	29.4	17.6
3	28.0	12.1

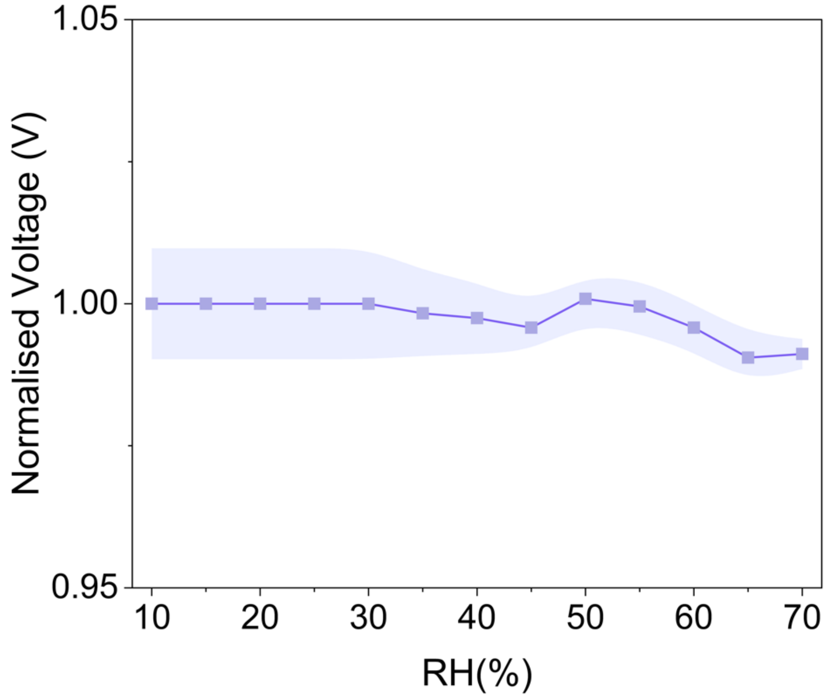

Supplementary Figure 15. Normalised voltage (to the maximum voltage) versus RH (10 RH%- 70 RH%) for the 2 h-HKUST-1 sample.

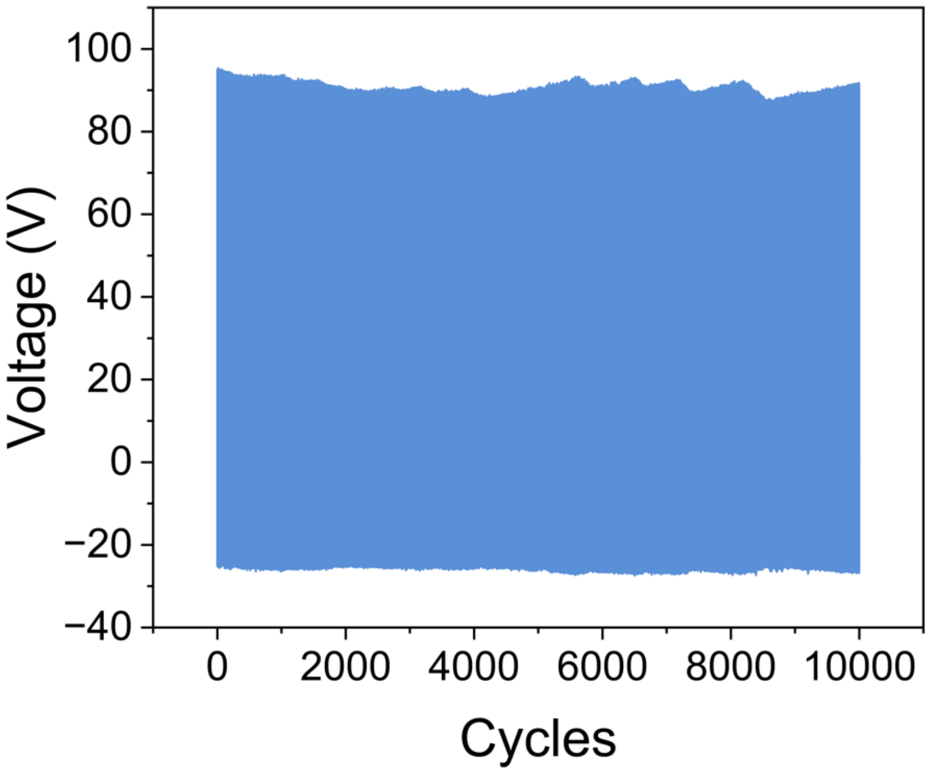

Supplementary Figure 16. Stability test of the 2 h-HKUST-1 device with ~10,000 cycles at high humidity, ranging from 70 RH% to 71.8 RH%.

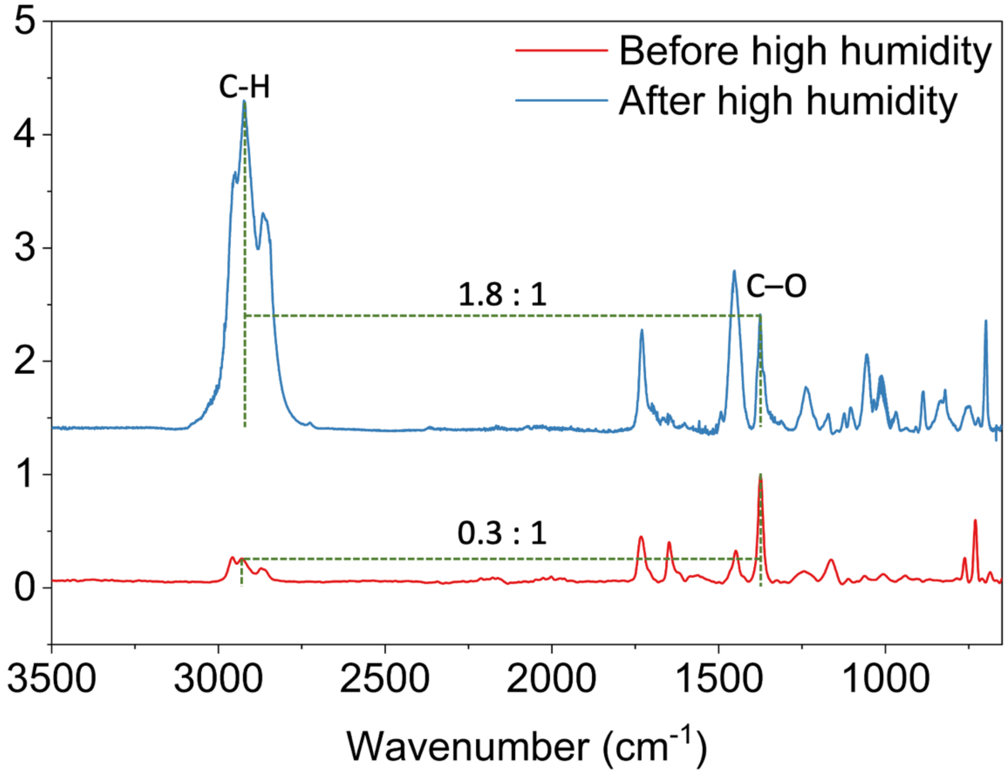

Supplementary Figure 17. ATR-FTIR spectra measured before and after TENG humidity test. The relative peak intensity of the designated bands was calculated relative to the C-O band at 1374 cm^{-1}. The C-H stretching mode of the BTC linker is identified at 2923 cm^{-1}.

Movie Clip

Supplementary Movie 1: Real-time testing of the 2 h-HKUST-1 TENG for illuminating 48 LEDs in the dark, under ambient conditions.

Supplementary references:

1 Khandelwal, G., Chandrasekhar, A., Maria Joseph Raj, N. P. & Kim, S. J. Metal–Organic Framework: A Novel Material for Triboelectric Nanogenerator–Based Self-Powered Sensors and Systems. *Adv. Energy Mater.* **9**, 1803581 (2019). https://doi.org:10.1002/aenm.201803581

2 Sun, J. *et al.* Functionalized wood with tunable tribopolarity for efficient triboelectric nanogenerators. *Matter* **4**, 3049-3066 (2021). https://doi.org:10.1016/j.matt.2021.07.022

3 Babu, A. *et al.* Facile Direct Growth of ZIF-67 Metal–Organic Framework for Triboelectric Nanogenerators and Their Application in the Internet of Vehicles. *ACS Sustain. Chem. Eng.* **11**, 16806-16817 (2023). https://doi.org:10.1021/acssuschemeng.3c05198

4 Velpula, M. *et al.* High-Performance MOF-303-Based Triboelectric Nanogenerators for Self-Powered Electronics and Road Safety Applications. *ACS Appl. Electron. Mater.* **6**, 7890-7897 (2024). https://doi.org:10.1021/acsaelm.4c01247

5 Wen, R. *et al.* Controllable design of high-efficiency triboelectric materials by functionalized metal—organic frameworks with a large electron-withdrawing functional group. *Nano Research* **15**, 9386-9391 (2022). https://doi.org:10.1007/s12274-022-4731-6

6 Khandelwal, G., Maria Joseph Raj, N. P. & Kim, S. J. Zeolitic Imidazole Framework: Metal–Organic Framework Subfamily Members for Triboelectric Nanogenerators. *Adv. Funct. Mater.* **30**, 1910162 (2020). https://doi.org:10.1002/adfm.201910162

7 Khandelwal, G., Maria Joseph Raj, N. P. & Kim, S.-J. ZIF-62: a mixed linker metal–organic framework for triboelectric nanogenerators. *J. Mater. Chem. A* **8**, 17817-17825 (2020). https://doi.org:10.1039/d0ta05067a

8 Hajra, S. *et al.* A new insight into the ZIF-67 based triboelectric nanogenerator for self-powered robot object recognition. *J. Mater. Chem. C* **9**, 17319-17330 (2021). https://doi.org:10.1039/d1tc04729a

9 Hajra, S. *et al.* Significant effect of synthesis methodologies of metal-organic frameworks upon the additively manufactured dual-mode triboelectric nanogenerator towards self-powered applications. *Nano Energy.* **98**, 107253 (2022). https://doi.org:10.1016/j.nanoen.2022.107253

10 Khandelwal, G., Maria Joseph Raj, N. P., Vivekananthan, V. & Kim, S.-J. Biodegradable metal-organic framework MIL-88A for triboelectric nanogenerator. *iScience* **24**, 102064 (2021). https://doi.org:10.1016/j.isci.2021.102064

11 Hajra, S. *et al.* A Green Metal–Organic Framework-Cyclodextrin MOF: A Novel Multifunctional Material Based Triboelectric Nanogenerator for Highly Efficient Mechanical Energy Harvesting. *Adv. Funct. Mater.* **31**, 2101829 (2021). https://doi.org:10.1002/adfm.202101829

12 Shaukat, R. A. *et al.* Ultra-robust tribo- and piezo-electric nanogenerator based on metal organic frameworks (MOF-5) with high environmental stability. *Nano Energy* **96**, 107128 (2022). https://doi.org:10.1016/j.nanoen.2022.107128

13 Shao, Z. *et al.* A Double-Helix Metal-Chain Metal-Organic Framework as a High-Output Triboelectric Nanogenerator Material for Self-Powered Anticorrosion. *Angew. Chem. Int. Ed.* **61** (2022). https://doi.org:10.1002/anie.202208994

14 Sarfudeen, S. *et al.* A Novel Mechano-Synthesized Zeolitic Tetrazolate Framework for a High-Performance Triboelectric Nanogenerator and Self-Powered Selective Neurochemical Detection. *ACS Appl. Mater. Interfaces.* **16**, 24851-24862 (2024). https://doi.org:10.1021/acsami.4c00454

15 Sarfudeen, S. & Panda, T. Core-shell nano-architectonics in metal organic framework for enhanced performance in triboelectric nano-generator and self-powered nitro-explosive/humidity sensor. *Chem. Eng. J.* **503**, 158519 (2025). https://doi.org:10.1016/j.cej.2024.158519

16 Gao, K. *et al.* 3D nanocrystalline metal–organic framework materials for the improved output performance of triboelectric nanogenerators. *Dalton Trans.* **52**, 444-451 (2023). https://doi.org:10.1039/d2dt03477h

17 Huang, C. *et al.* Enhancement of Output Performance of Triboelectric Nanogenerator by Switchable Stimuli in Metal–Organic Frameworks for Photocatalysis. *ACS Appl. Mater. Interfaces.* **14**, 16424-16434 (2022). https://doi.org:10.1021/acsami.2c01251

18 Chen, J. *et al.* Metal-Ion Coupling in Metal–Organic Framework Materials Regulating the Output Performance of a Triboelectric Nanogenerator. *Inorg. Chem.* **61**, 2490-2498 (2022). https://doi.org:10.1021/acs.inorgchem.1c03338

19 Zhang, Y. *et al.* Molecular-functionalized metal-organic frameworks enabling contact-electro-catalytic organic decomposition. *Nano Energy* **111**, 108433 (2023). https://doi.org:10.1016/j.nanoen.2023.108433
