# Supplementary material for: Robust triboelectric energy harvesters engineered from electrochemically deposited films of HKUST-1 polycrystals
Source: Commun Chem. 2026 Feb 25;9:144. doi: 10.1038/s42004-026-01949-0 (PMC13046843; doi:10.1038/s42004-026-01949-0)
Supplement: Supplementary file 3 — Description of Additional Supplementary Files [file 42004_2026_1949_MOESM3_ESM.pdf]

# Description of Additional Supplementary Files:

**File name:** Supplementary Movie S1
**Description:** Real-time testing of the 2 h-HKUST-1 TENG for illuminating 48 LEDs in the dark, under ambient conditions.

**File name:** Supplementary Data 1
**Description:** Numerical data set for all figures except Figure 3a.

**File name:** Supplementary Data 2
**Description:** Numerical data set for Figure 3a.
